# Characterizing population-based health care needs at the departement level in France: Ulysse Rodts

**DOI:** 10.1093/eurpub/ckac129.690

**Published:** 2022-10-25

**Authors:** U Rodts, T Ngo, E Ros, L Millet, A Malâtre-Lansac

**Affiliations:** 1 KanopyMed, Clapiers, France; 2 Institut Montaigne, Paris, France

## Abstract

Aligning health care spending with population needs is a goal shared by many public health and health care systems. However, most modelling approaches have proven deceptive and ineffective. We propose a novel data-driven, population-based approach to help policymakers explore the discrepancies between spending and needs in France. We leveraged several national open data sources covering demographics, social deprivation, epidemiology, environment, health-related behaviors, and all-payer health care spending (hospital inpatient, prescription medicines, ambulatory events, and dental care). We classified the French “departements” (hereafter counties) into clusters that are homogeneous in terms of health care needs, based on a multidimensional framework. Then, we calculated all-payer per capita health care spending to analyze its within- and between-cluster variation. Based on these findings, we designed a web-based, interactive mapping tool dedicated to French policymakers and payers. The analysis shows 7 clusters of French counties differing in terms of health care needs and spending. The higher-needs/lower-spending and lower-needs/higher spending clusters suggest considerable room for improvement through a regional distribution of spending at least partially based on needs. Most interestingly, the data we used is publicly available, but policymakers lack expertise and time to undergo such analyses themselves. We plan to develop a dynamic and more granular version of the tool to allow policymakers to accurately design and evaluate health care policies.

